# The potential immunomodulatory role of human milk oligosaccharides in prevention of viral infections and development of asthma in early life

**DOI:** 10.3389/fimmu.2025.1572787

**Published:** 2025-06-25

**Authors:** Vera Rijks, Marit Zuurveld, Johan Garssen, Atanaska I. Kostadinova, Linette E.M. Willemsen

**Affiliations:** ^1^ Department of Pharmaceutical Sciences, div. Pharmacology, Utrecht University, Utrecht, Netherlands; ^2^ Global Center of Excellence Immunology, Danone Global Research & Innovation Center, Utrecht, Netherlands

**Keywords:** asthma, viral infections, human milk oligosaccharides, short chain fatty acids, mucosal immunity

## Abstract

Around 10% of the Western population is diagnosed with asthma, and this percentage is only expected to increase in the coming years. Allergic asthma often develops during early infancy and is characterized by chronic pulmonary type 2 inflammation and airway hyperresponsiveness. Severe viral infections in early life are thought to be a risk factor for allergic asthma. The most common causes of severe viral infections in early life are respiratory syncytial virus (RSV) and rhinovirus (RV). How viral infections in early life are related to the later development of asthma is not yet known, but the pathophysiology of RSV/RV infection and asthma overlap in several areas. RSV and RV are both able to induce type 2 immunity which may contribute to the development of allergic asthma which is driven by type 2 responses against airborne allergens such as house dust mites. In early life, infants’ intestines, microbiome and immune function need to mature, and breastfeeding helps to facilitate these major steps in development. Human milk oligosaccharides (HMOs) are the third largest component of human milk and have been shown to promote the development and function of the infant microbiome and may have a beneficial effect on immune maturation by promoting type 1 and regulatory immune responses. In addition, they can stimulate epithelial barrier integrity and directly interact with glycan receptors. Certain bacteria in the gut can metabolize HMOs into short-chain fatty acids (SCFA), which can exert beneficial anti-inflammatory effects locally in the gut or systemically and help maintain barrier properties and immune homeostasis. HMOs and SCFA could have protective effects on both the immune pathways in allergic asthma and viral infections. This review describes the molecular and immunomodulatory mechanisms by which different HMOs and SCFA may help defend against viral infections and also protect against allergic asthma.

## 1 Introduction

Asthma affects both adults and children and is one of the most common noncommunicable diseases worldwide (1). It is characterized by symptoms such as coughing, wheezing, shortness of breath, and chest tightness, caused by chronic inflammation in the lungs (2). The incidence of asthma has steadily increased in the Western world over the past few decades. Around 10% of the Western population is diagnosed with asthma, and this percentage is only expected to increase in the upcoming years (1, 3). Asthma can decrease the quality of life and places a financial burden both on asthmatic individuals and their families, as well as on society. Several different phenotypes of asthma exist, such as allergic asthma, non-allergic asthma, late-onset asthma, obesity-related asthma, neutrophilic asthma, and exercise-induced asthma (4). Allergic asthma is the most common type of asthma and will therefore be the focus of this review (5). The etiology of allergic asthma is still not fully understood, but it is generally thought to consist of multiple factors. These factors include genetic factors, and many different genes associated with asthma have been identified. No single gene or combination of genes can, however, predict whether a person will develop asthma or not. Additionally, environmental triggers, such as air pollution, life style, dust mites, or pollen, are suggested to play a role in the etiology of asthma (1, 6).

Beyond these contributing factors, severe respiratory viral infections in early life are thought to be a risk factor for asthma (7–9). Respiratory syncytial virus (RSV) and rhinovirus (RV) are the most common cause of severe respiratory infections in infants (10). Around 20%-30% of infants (<1 year) suffer from bronchiolitis caused by RSV or RV. 2% to 3% of children are hospitalized before the age of 1, with RSV responsible for 50%-80% and RV responsible for 5%-25% of bronchiolitis-related hospitalizations (11). Proper viral defense in early life is essential, as it can protect children from the possible severe outcomes of respiratory infections, and can even lower the risk of developing allergic asthma later in life.

Breastfeeding is the gold standard in feeding for infants. Human milk is rich in nutritional factors and contains many different bioactive components, such as antibodies, immune cells, microbes, cytokines and chemokines, and growth factors (12). Human milk furthermore contains human milk oligosaccharides (HMOs), a large group of non-digestible oligosaccharides (NDOs). HMOs are the third largest solid component of human milk. They are present in a concentration of 20–25 g/L in the colostrum and 5–15 g/L in mature human milk (13, 14). HMOs are a diverse group of structures; currently more than 200 HMO structures have been identified (14, 15). HMOs can be fermented by beneficial bacteria in the gut and thus help to develop the microbiome, while also supporting gut and immune maturation (16). Some of the health benefits observed in breastfed children are thought to be mediated at least in part by HMOs. Such health benefits are, for example, fewer airway infections in early life and a lower risk of allergic asthma later in life (13). Clinical studies show a protective effect of breastfeeding in development of asthma later in life. Recent meta-analyses show a general effect of breastfeeding on reducing the risk of asthma in children up to 12 and 18 years of age (17, 18). There is no scientific consensus on which component(s) of human milk are responsible for this effect. Studies on various components of human milk have been done and for example show an effect of the TGFβ levels present in human milk or the composition of the milk microbiome on the development of allergies and asthma (19, 20). Studies remain inconclusive however and the study of other components of human milk, such as HMOs, could further explain the possible protective effect of breastfeeding on the development of asthma. HMOs can potentially exert protective effects on viral infections and asthma in several ways, which are described in detail in this review.

Human milk contains many different types of HMOs. When breastfeeding is not possible, formula milk products are used. These are however mainly based on ingredients derived from cow’s milk, which do not contain HMOs. Over the years, formula milk has been enriched with NDO mixtures, such as galacto-oligosaccharides (GOS) and long-chain fructo-oligosaccharides (lcFOS). NDO have been shown to support *bifidobacterium* and *lactobacillus* spp. colonization almost similar to human milk (21). However, even with these substantial improvements, formula milk still does not resemble the complexity of human milk.

Food and Drug Administration (FDA)-approved synthetically produced HMO structures became available for large-scale industrial production relatively recently. The addition of specific HMOs structures to formula milk may help to further improve the development of the infant’s microbiome and maturation of the gut and immune system, and therefore potentially have beneficial, protective effects against respiratory viral infections and the development of allergic asthma (16).

In this review, we explore the possible mechanistic relation between viral infections in early life and the development of asthma. Additionally, studies suggest that HMOs can protect against both severe respiratory viral infections and the development of asthma. We first explore the mechanisms through which specific HMOs may exert protective effects during viral infections. This includes their role in preventing viral adhesion, modulating the microbiome and epithelial barrier, and influencing both innate and adaptive immune responses through interactions with various receptors. We also review relevant pre-clinical and clinical evidence. Following this, we examine how HMOs might contribute to asthma prevention, using a structure similar to the outline on viral infections. Finally, we highlight the overarching (immunomodulatory) mechanisms that link direct effects of HMO and their indirect effects via bacterial fermentation products in viral infections and asthma development.

## 2 Pathophysiology of allergic asthma

### 2.1 Sensitization/induction phase

**Figure 1:**
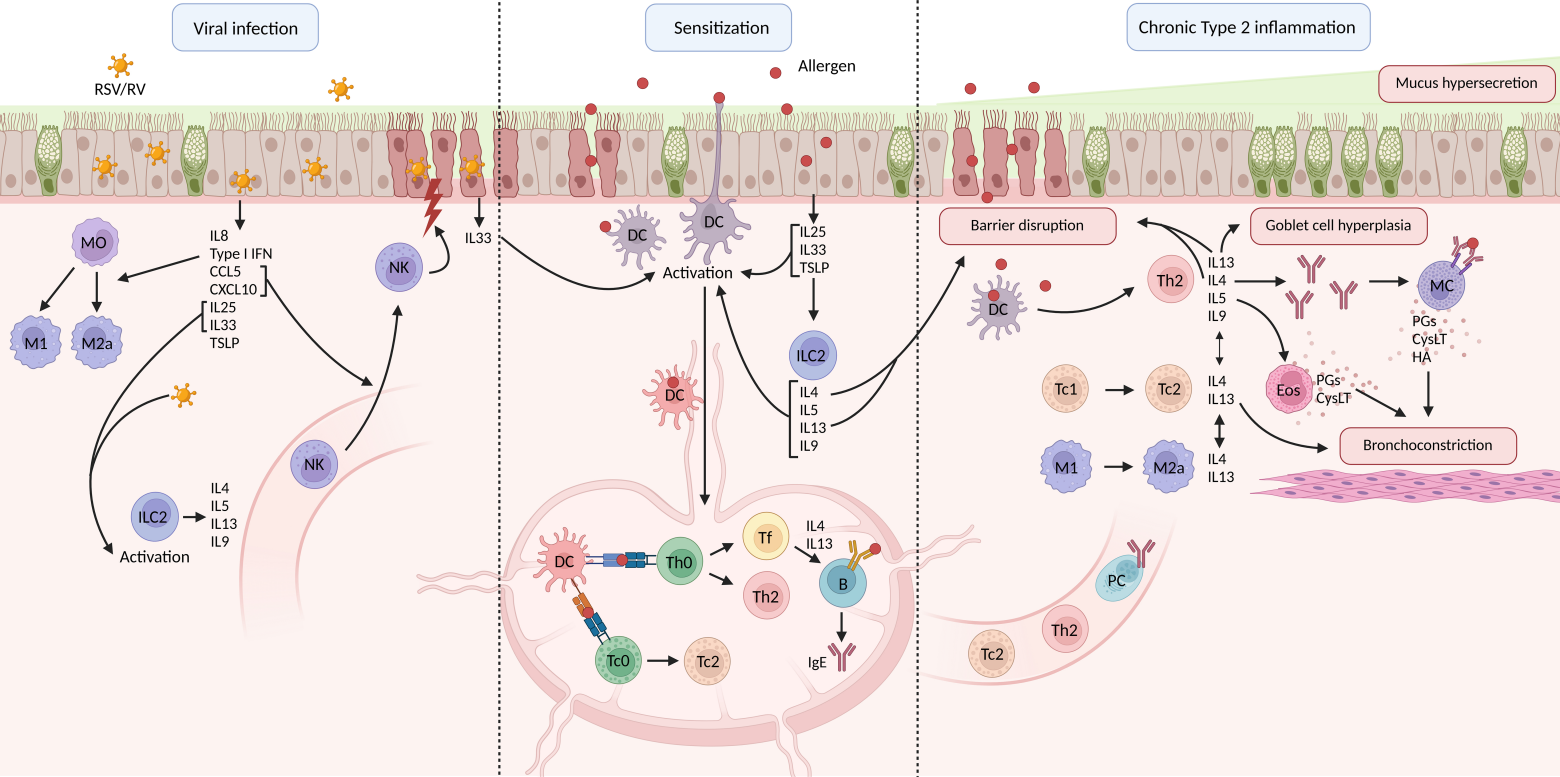
Schematic overview of respiratory viral infection, allergic asthma pathophysiology and chronic type 2 inflammation. B, B cell; CysLT, cysteinyl leukotrienes; DC, dendritic cell; Eos, eosinophil; HA, histamine; Ig, immunoglobulin; IL, interleukin; ILC, innate lymphoid cell; M, macrophage; MC, mast cell; MO, monocyte; NK, natural killer cell; PC, plasma cell; PG, prostaglandin; RSV, respiratory syncytial virus; RV, rhinovirus; Tc, cytotoxic T cell; Th, T helper cell; TSLP, thymic stromal lymphoprotein (created in BioRender.com).

Allergens can activate bronchial epithelial cells (BECs) via pattern recognition receptors (PRRs) (2). BECs secrete thymic stromal lymphopoietin (TSLP), interleukin (IL)25, IL33, C-C motif ligand 2 (CCL2), and CCL20 in response to allergen binding (22, 23). Dendritic cells (DCs) are activated by IL33 and TSLP or by direct allergen binding. Furthermore, IL25 and IL33 attract and activate group 2 innate lymphoid cells (ILC2s) (24). ILC2s produce IL4, IL5, IL9, and IL13, promoting local inflammation and DC maturation (24, 25). ILC2s are generally activated before allergen-specific T helper (Th) 2 cells are recruited, making them an important source of type 2 cytokines in the sensitization phase in allergic asthma (**Figure 1**) (5, 26).

Activated DCs take up the allergen and migrate to the mediastinal lymph nodes (medLNs). Here, they present the processed allergen via the major histocompatibility complex II (MHCII) to the T cell receptor (TCR) on naïve T cells. This, combined with the stimulation of co-stimulatory molecules, induces the allergen-specific naïve T cells to differentiate into Th2 cells (3, 5, 27, 28). Th2 cells home to the lungs, where they secrete IL4, IL5, IL13, and granulocyte-macrophage colony-stimulating factor (GM-CSF) (3, 29–31). IL4 secreting follicular helper (Tfh) also develop from naïve T cells, which instruct allergen-specific B cells in the medLN towards Immunoglobulin (Ig) E isotype switching (**Figure 1**) (32).

In the tissue, IL4 promotes IgE secretion by plasma cells and induces the upregulation of high-affinity IgE receptor (FCϵR) on mast cells (33–35). IL13 mainly mediates mucus hypersecretion and airway hyperresponsiveness, while IL5 plays a role in the migration and activation of eosinophils in the lung tissue (**Figure 1**) (36).

Recent studies have shown the possible involvement of type-2 CD8+ cytotoxic T cells (Tc2) in the pathophysiology of allergic asthma as well. These effector Tc are also generated within the medLN, but is driven by antigen presentation via MHCI instead of MHCII (37). Tc2 are capable of producing type 2 cytokines and Tc1 cells can be skewed towards Tc2 under inflammatory conditions, which is promoted by IL33 signaling (38–41).

### 2.2 Effector/symptom phase

Upon secondary exposure, allergens bind IgE on the surface of mast cells, leading to crosslinking of the FCϵR receptors, and degranulation (32, 42). Inflammatory mediators such as histamine (HA), prostaglandins (PGs), and cysteinyl leukotrienes (cysLT) are released and induce bronchoconstriction, mucosal edema, and excessive mucus secretion (3, 32, 42). IL9 is also known to induce excessive mucus secretion (43). The characteristics of chronic asthma are induced by the release of IL4, IL5, and IL13 by Th2 cells (3, 5, 32). These cytokines mediate eosinophilic airway inflammation, mucus hypersecretion, goblet cell hyperplasia, and proliferation of IgE-producing plasma cells (2, 32, 44). Tc2 furthermore play a role in asthma exacerbations as well, as demonstrated by an increase in Tc1 to Tc2 skewing observed in patients experiencing an asthma exacerbation (**Figure 1**) (41).

Macrophages also participate in the chronic inflammation in allergic asthma (5, 45). M1 macrophages are generally considered to be the pro-inflammatory macrophages and M2 macrophages the anti-inflammatory macrophages (46). However, the presence of M2a macrophages contributes to allergic inflammation as well (47). The M2a phenotype is induced by IL4 and IL13 and M2a secrete high levels of IL1β, IL6, IL33, TSLP, CCL2, CCL17, and CCL22 upon activation (46, 47). These cytokines and chemokines are known to participate in the activation of Th2, stimulation of ILC2s, and promote eosinophilia (45). Moreover, M2a macrophages might also participate in the tissue remodeling observed in chronic asthma, through transforming growth factor β (TGFβ) and platelet-derived growth factor (PDGF). This needs however yet to be confirmed in humans (45, 48, 49).

## 3 Possible role of respiratory viral infection in early life on the development of allergic asthma

Severe respiratory viral infections can progress into bronchiolitis, which is characterized by symptoms such as wheezing and shortness of breath (50). The connection between viral-induced bronchiolitis and asthma has been suggested by several epidemiological studies. The most common viral causes of bronchiolitis and wheezing in children are RSV and RV (10). RSV is an enveloped negative-strand RNA virus, while RV is a large family of non-enveloped RNA viruses, and is divided into 3 clades A, B, and C (9, 51). Several longitudinal studies show an increased risk for the development of asthma after severe infection with RSV and/or RV in early life. Sigurs et al., for example, showed that children who suffered from RSV-induced bronchiolitis during the first three years of life had a 30% risk of developing asthma before the age of 7, compared to only 3% in the control group, who had not had RSV infection (52). More recently, a much larger study including 740.418 children in Scotland, confirmed these findings, showing that children who had suffered from a severe RSV infection in early life had a three-fold higher risk for asthma-related hospitalizations and a two-fold higher asthma medication usage at an average age of 10.6 years (interquartile range [IQR]: 6.9‐14.6) (7). Similar observations are described for RV infections. A study done in Finland, for example, showed an increased risk of developing asthma at age 8 if children had suffered from RV-induced wheezing in early life (odds ratio, 13; 95% CI, 4.3-41) (8, 9). The epidemiological data point to a connection between RSV and RV infection in early life and the later development of asthma. However, it is currently unknown whether viral infections are a causal factor in the development of asthma. In this review, we will dive into the cellular mechanisms of viral infection and their possible connection to the development of allergic asthma, and the possible protective role of HMOs.

### 3.1 Pattern recognition receptor activation upon viral infection

Various immune responses are initiated upon respiratory viral infection. The epithelial barrier is the first line of defense where pulmonary epithelial cells (ECs) recognize viral pathogen-associated molecular patterns (PAMPs) through PRRs (53). Important PRRs for virus recognition include the TLRs (e.g. TLR3, TLR7, TLR8, and TLR9), the retinoic acid inducible gene-I (RIG-I)-like receptors (RLRs), the nucleotide oligomerization domain (NOD)-like receptors (NLRs), and the absent in melanoma 2 (AIM2)-like receptors (ALRs) (54–57). Recognition of PAMPs by PRRs on pulmonary ECs activates signaling pathways that eventually result in the production of type I interferons (IFNs), pro-inflammatory cytokines (IL6, tumor necrosis factor α (TNFα), granulocyte colony stimulating factor (G-CSF), GM-CSF) and chemokines (IL8, C-X-C chemokine ligand 10 (CXCL10), CCL5), and assembly of inflammasomes. This innate immune response is the first response upon viral encounter and will subsequently activate the adaptive immune response (**Figure 1**) (50).

### 3.2 Innate immune responses during viral respiratory infections

During infection with RSV or RV, airway inflammation and airway remodeling can take place, compromising the integrity of the epithelial barrier (58–60). The remodeling of the airways induced by RSV infection starts with the cytokines produced by the epithelial cells in response to the viral infection, such as type I IFNs. Several underlying structural and immune cells are activated and/or recruited to the lungs, such as lymphocytes and eosinophils, which can locally produce inflammatory mediators. Fibroblasts and smooth muscle cells in the lungs respond to the inflammatory mediators by increasing their proliferation and matrix deposition, eventually leading to airway remodeling (9, 61). Furthermore, the integrity of the epithelial barrier is compromised by RSV and RV infection, due to apoptotic cell death of ECs in response to the infection and through degradation of the tight junctions (TJs) (59, 62, 63). In relation to allergy, a decrease in barrier integrity allows aeroallergens to easily cross the epithelial barrier and interact with DCs (60). Moreover, lung ECs can produce the alarmins IL25, IL33, and TSLP in response to viral infection, which also play an important role in the development of asthma (64, 65). Lastly, RSV can activate ILC2s, which are further stimulated by IL25 and IL33 released by damaged ECs (66). ILC2s release IL4, IL5, IL9, and IL13, enhancing a type 2 immune response and therefore potentially also promoting the development of allergic asthma (24, 25, 66).

RSV infections are characterized by a strong neutrophilic response, as observed by a sharp increase in the number of neutrophils both systemically and in the respiratory tract (67). RSV can directly interact with and infect neutrophils in severe infections (68). Additionally, some studies suggest that during RSV-induced bronchiolitis, eosinophils are recruited to the lungs, with higher levels of eosinophils in the lungs being associated with more severe infection (69–71). Other immune effects are associated with severe RSV infection as well. For example, low levels of plasmacytoid DCs (pDCs), and on the other hand increased numbers of conventional DCs (cDCs) in the blood are associated with the development of RSV-induced bronchiolitis (72). cDCs exert a pro-inflammatory phenotype promoting activation of T cells, natural killer T cells, and natural killer (NK) cells in the lower respiratory tract (73). NK cells play a critical role in the innate immune response against viral infections, as they can kill infected cells and secrete cytokines to recruit and activate other immune cells (74). NK cells are recruited from the bloodstream by inflammatory mediators (such as CXCL8, CCL5, CXCL10) produced by ECs in response to viral recognition (75, 76). NK cells express both activating and inhibiting receptors (74, 77, 78). Under normal conditions, all body cells express MHC I molecules which inhibit the cytotoxic activity of NK cells (78, 79). When a cell is infected with a virus, its expression of MHC I can be downregulated, which prevents the detection of the infected cells by CD8+ cytotoxic T cells. On the other hand, decreased MHCI expression activates the NK cell to kill the infected cell (78). Proper NK cell function is important for viral defense. The killing of infected cells by NK cells happens through various mechanisms, such as the release of cytotoxic granules, death receptor mediated apoptosis, or antibody-dependent cellular toxicity (50). Furthermore, NK cells secrete various cytokines, such as IFNγ, TNFα, and GM-CSF, which can activate other immune cells (78). Interestingly, an NK cell deficiency has been observed in children suffering from RSV infection (80). In addition, animal studies in mice with pre-existing NK deficiency have reported that NK cell deficiency during RSV infection induced skewing towards a Th2 response, which was dependent on IL25 secreted by lung ECs (80). Such insights suggest a possible link between NK deficiency during RSV infection and predisposition to a Th2 prone immune response.

Macrophages are of great importance for viral defense as well, as they are one of the first immune cells to encounter viruses in the lung (81). Recognition of viral particles by macrophages induces the release of large amounts of cytokines and chemokines, recruiting more inflammatory cells, such as monocytes, towards the site of infection (81, 82). BECs can also be infected by a virus, triggering the production of IFNα/β, which activates macrophages as well (83). Once present in the lung, monocytes can differentiate into different subsets of macrophages. At the start of viral infections, M1 macrophages are needed, while the number of M2a macrophages increases during the course of the infection to promote tissue repair (82). In general, infants have a bias towards M2 skewed macrophages, resulting in a lower production of pro-inflammatory cytokines, and thus possibly a reduced ability to clear a viral infection (84, 85). In mice, however, lung pathology caused by RSV infection was reduced when M2 differentiation was induced, showcasing the protective effect of M2 macrophages (86).

### 3.3 Adaptive immune response during viral respiratory infections

Secretion of cytokines by lung ECs, such as the type I IFNs, is needed for the onset of the adaptive immune response. Such cytokines lead to the activation of DCs, NK cells, and macrophages, the enhancement of activities of various lymphocytes, and the stimulation of antibody production. DCs are impacted by type I IFNs in various ways. Type I IFNs stimulate the differentiation of bone marrow derived monocytes into DCs, induce the expression of chemokine receptors such as CCR7 for migration to the lymph nodes and stimulate antigen presentation and the expression of costimulatory molecules required for T cell activation (50, 87–89). In children with severe RSV infection, Th2 skewing can be observed, instead of a Th1 response that normally occurs in viral infections (90).

Clearance of RSV infection is most likely mediated by plasma cells which produce neutralizing antibodies and by recruited Tc cells that directly kill infected cells (91). All types of circulating T cells are decreased in severe RSV infection (92, 93). The low T cell counts in RSV-infected infants may be the result of immune evasion strategies of RSV. Fas-ligand and caspase-1 levels are elevated in RSV-infected individuals, potentially inducing T cell apoptosis (94). Moreover, programmed cell death 1 (PD-1) protein is elevated in CD8+ T cells in RSV lower respiratory tract infection (LRTI), inhibiting pro-inflammatory cytokine production in activated T cells and further suppressing the T cell response (95). The generation of memory T cells from CD8+ T cells might also be compromised by the activation of mammalian target of rapamycin (mTOR) by RSV. Higher expression of mTOR has been found in the lungs of infants with RSV-induced bronchiolitis, potentially reducing the number of memory T cells and thus lowering the protection against reinfection (96). In addition, an association is found between lower levels of systemic IFNγ (a typical marker of type 1 response) and an increased risk of RSV-induced bronchiolitis and the need for ventilation (97). IFNγ in the lungs exerts a protective effect during viral infection and lower levels of IFNγ in the lungs are associated with more severe disease (98, 99). Type 2 responses can also be induced by RSV infection. Several type 2 markers, both systemically and locally in the lungs, are associated with disease severity (71, 100). A high IL4/IFNγ ratio, indicating a type 2 bias, is associated with severe RSV-induced bronchiolitis (90). Clinical evidence supports the hypothesis that a type 2 skewed response is associated with severe RSV infection, as studies have found type 2 cytokine profiles, rather than type 1 cytokine profiles, in children hospitalized with severe RSV (100). Lastly, systemic mature and precursor B cells are present in higher numbers in RSV LRTI, and IgA, IgG, and IgM antibodies in response to the F and G glycoproteins of RSV are present in the lungs of infants with RSV LRTI (101–103). An IgE response against the RSV R and G glycoproteins can also be initiated and is associated with severe disease (104). Children with high levels of IgE exhibited worse symptoms and prolonged disease and also showed higher eosinophil counts (71, 105).

The mechanism by which RV infection could contribute to the development of asthma is less clear than for RSV infection. RV does not have a cytopathic effect on the lung ECs but is still able to compromise the epithelial barrier integrity through degradation of the TJs (59, 106, 107). Furthermore, similar to RSV infection, RV infection is suggested to be able to induce a type 2 response, which could contribute to the subsequent development of asthma (65, 108, 109). Further evidence that viral infections in early life contribute to the development of asthma, comes from studies treating viral-induced wheezing in children. These studies show that treating children suffering from RV-induced wheezing with oral corticosteroids decreased the initiation of the use of asthma controller medication within 5 years by 30%-40% (110, 111). No reduction in the development of asthma was however observed in a similar study treating children suffering from RSV infection with palivizumab, an antibody directed against RSV (112, 113). Viral infection in early life and the subsequent development of asthma could be related via various mechanisms. The nature of this relation, and whether viral infections are a causal factor, have however yet to be elucidated. Research into the molecular mechanisms of viral infection and allergic asthma, as well as large cohort studies could provide valuable information to answer this question.

## 4 What are human milk oligosaccharides?

**Figure 2:**
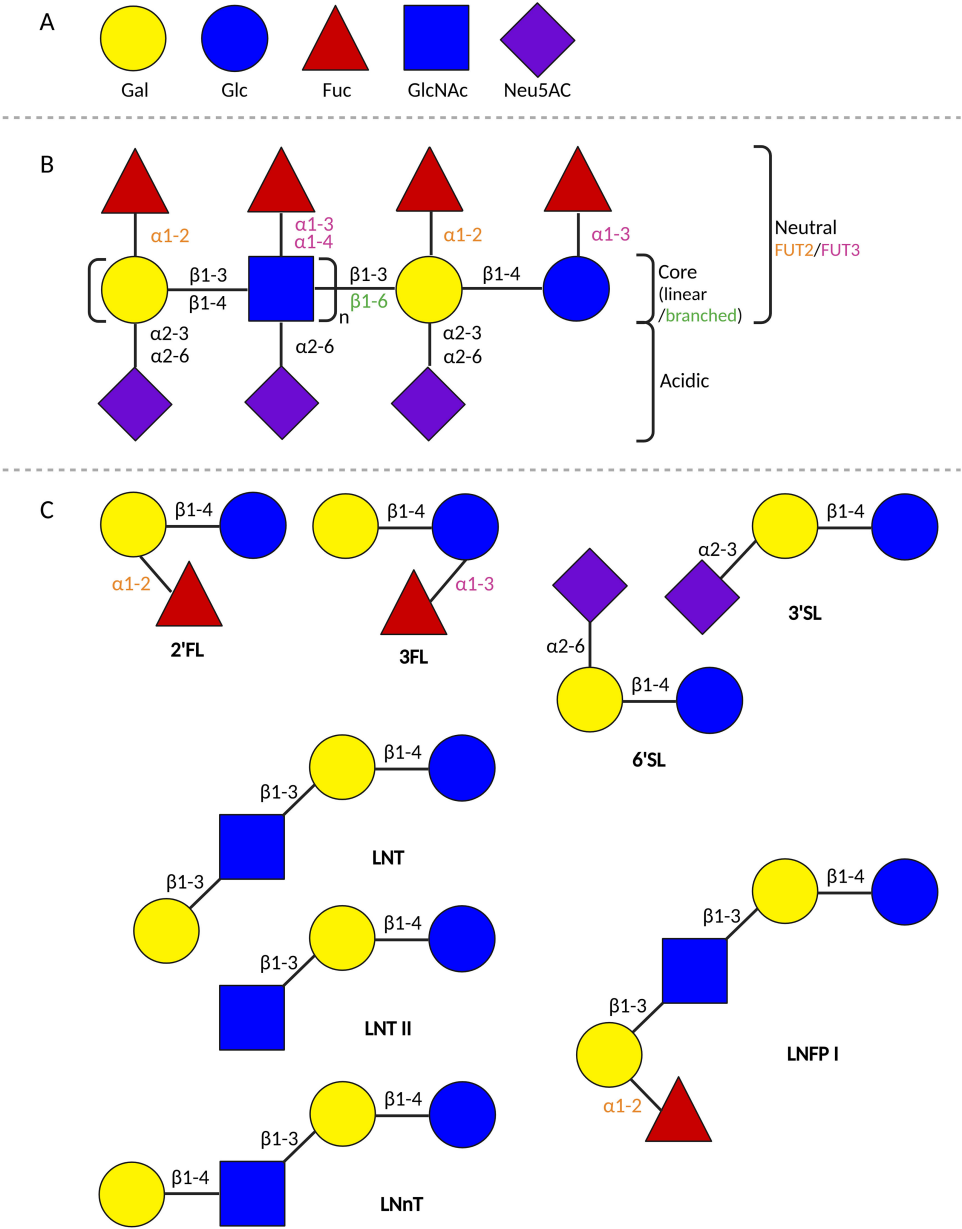
Schematic overview of HMOs. **(A)** The 5 building blocks of all HMOs. **(B)** Schematic representation of the building scheme of HMOs. **(C)** Schematic representation of the composition of different HMOs structures. 2’FL, 2’-fucosyllactose; 3FL, 3-fucosyllactose; 3’SL, 3’-siallylactose; 6’SL, 6’-siallylactose; Fuc, fucose; FUT, fucosyltransferase; Gal, galactose; Glc, glucose; GlcNAc, n-acetylglucosamine; LNFP I, lacto-N-fucopentaose I; LNT, lacto-N-tetraose; LNnT, Lacto-N-neotetraose; LNT II, Lacto-N-triose; Neu5AC, sialic acid (created in BioRender.com).

Human milk is a biologically complex fluid comprised of many components. One of the most abundantly present solid ingredients in human milk are the HMOs. Within this complex group of NDOs, many different HMO structures have been identified so far (14, 15, 114). The various HMO structures consist of 3 to 23 monosaccharide units and are created from the same five building blocks: glucose (Glc), galactose (Gal), fucose (Fuc), n-acetylglucosamine (GlcNAc), and sialic acid or n-acetylneuraminic acid (Neu5Ac) (13, 14, 16, 115). In the mammary gland, Glc and Gal are linked with a β1–4 glycosidic linkage by lactose-synthase to form lactose. Lactose forms the basis at the reducing end of all known HMOs structures. The HMOs structures are further elongated by the addition of Gal and/or GlcNAc units to lactose with a β1–3 or a β1–6 glycosidic linkage, or through fucosylation and sialylation. During fucosylation, Fuc is added to Gal through α1–2 linkage or to Glc/GlcNAc through α1-3/4 linkage. In sialylation, Neu5Ac is added to Gal through α2-3/6 linkage, or to GlcNAc through α2–6 linkage (13, 14, 115). Sialylation results in acidic HMOs, while all others are considered neutral (**Figure 2**) (13).

The composition of HMOs in human milk varies per person and is dependent on several factors, such as the lactation stage, maternal diet, and maternal phenotype (116, 117). The maternal phenotype results from genetic polymorphisms in the Secretor (Se) and Lewis (Le) genes, encoding the fucosyltransferase (FUT) -2 and FUT3 enzymes, respectively. These enzymes determine the fucose linkages that a lactating person can create (14, 16, 118, 119). Four maternal phenotypic variants can be distinguished: Se+Le+, Se-Le+, Se+Le-, and Se-Le- (119). Se+Le+ allows the lactating person to make α1-2/3/4 fucose linkages (e.g. 2’fucosyllactose (2’FL), 3-fucosyllactose (3FL)), Se-Le+ enables α1-3/4 (e.g. 3FL, lacto-N-fucopentaose (LNFP) II, III), Se+Le- α1-2/3 (e.g. 2’FL, 3FL), and Se-Le-, α1-3 (e.g. 3FL, LNFP III) (14).

HMOs have been linked to several biological functions in early life development, such as supporting the establishment of the gut microbiome in newborns. The intestinal microbiome is important for general health and for the proper development and functioning of the immune system (120). Infants are born with an immature microbiome that should mature during the first years of life, and reach a composition comparable to healthy adults around the age of 2 to 5 years (121). HMOs play an important part in the establishment of the gut microbiome. HMOs act as prebiotics and favor the colonization of commensal bacteria, such as *Bifidobacterium* and *Lactobacillus* species (122). The microbiota of breastfed infants specifically includes *Bifidobacterium longum* subspecies *infantis (B.infantis)*, which is specialized and highly efficient in the metabolization of HMOs (123).

Studies have shown that the microbiome of formula-fed children is different compared to breastfed children (120). In one study the composition of the microbiome was compared between breastfed infants, formula milk supplemented with 2’FL and lacto-N-neotetraose (LNnT) fed infants, and a control group fed formula milk without the supplements. The microbiome of the children that were fed the formula milk supplemented with 2’FL and LNnT was more similar to breastfed children, as was observed previously for formula milk containing more general NDOs (21, 124). The microbiome of the formula-fed infants without the HMOs supplements contained higher levels of *Escherichia Coli* and *Clostridium difficile* (124). These results suggest that supplementing formula milk with HMOs may contribute to infant’s health, as a healthy microbiome is important for proper immune functioning (120).

HMOs are found to have more functions and effects on the body besides supporting the development of a healthy intestinal microbiome. For example, HMOs can enhance the gut epithelial barrier and both directly and indirectly interact with the immune system (14, 16, 112). Neutral and acidic HMOs have been observed to cross the epithelial barrier in the gut, and neutral HMOs are actively transported over the epithelial barrier as well (125). These observations suggest a systemic availability of HMOs, which is further supported by the detection of HMOs in the urine and peripheral blood of infants (126). HMOs have also been detected in the amniotic fluid. Amniotic fluid can enter the lungs of the fetus, thus exposing the lungs to the HMOs present in the amniotic fluid. HMOs might exert a direct effect on the lungs during gestation and possibly shortly after birth (127).

Furthermore, while HMOs are indigestible for the infant, various bacteria can digest HMOs into SCFA, which also support the barrier integrity of the gut and can modulate immune responses (14, 16). SCFA have been detected in the blood of infants as well (128). The systemic availability of SCFA is relatively low: 100 µM in serum for both humans and mice, of which acetate makes up 80%. The concentration that reaches the lungs is therefore, also low (129–131). Studies, however, show that SCFA have systemic effects despite the low systemic availability. Trompette et al. for example administered propionate in the drinking water of mice, which resulted in decreased eosinophil recruitment to the lungs, decreased goblet cell hyperplasia and decreased mucus production, via imprinting the development of tolerogenic DC in the bone marrow (132). The influence of HMOs and SCFA on viral infections and the development of allergic asthma has been studied and will be discussed in detail in the following sections.

## 5 Protective effects of HMOs during viral infection

### 5.1 Inhibition of viral adhesion to cells

Viruses bind cell surface receptors to enter cells and replicate (133). HMOs can intervene with the adhesion of the virus to the cell in two different ways: 1) as HMOs resemble glycan receptors found on epithelial cells they can directly interact with the virus and act as soluble decoy receptors (133, 134). Viruses that bind the HMOs will not be able to bind a glycan receptor on a cell, and therefore will not be able to infect and replicate; 2) HMOs can also bind to receptors on ECs themselves, thereby competing with viruses and blocking viruses from binding and entering cells (133, 134). That HMOs can reduce the viral load in both ECs was for example shown in an *in vitro* study, where 2’FL and 3’SL downregulated the viral load of RSV in 16HBE cells, while LNnT and 6’SL downregulated viral load of influenza A (135).

Not all HMOs can bind viruses or epithelial cell receptors. Rather, this function is dependent on the structure of the HMOs. Fucosylated HMOs most closely resemble the glycan receptors found on cells and thus most often fulfill the role of preventing viral adhesion (14). A large percentage (35% to 50%) of the HMOs found in human milk is fucosylated, indicating a potential biological function in viral defense (14).

Furthermore, direct interaction of HMOs with intestinal ECs (IECs) can modulate their gene expression, potentially resulting in changes in surface glycans expression, as well as in other cellular responses, such as cytokine production (136). Such effects of HMOs may play a role in the responses upon encountering invading viral pathogens. The modification of surface glycans expression could be a plausible mechanism by which HMOs interfere with the adhesion of pathogens at the lung epithelial barrier site and could prevent infection. Since low concentrations of HMOs become systemically available, it can be hypothesized that HMOs modulate lung ECs gene expression, and cytokine secretion in the *in vivo* situation (126). However, no experimental data is available to confirm this currently.

### 5.2 Modulation of the microbiome and epithelial barrier

Proper development of the microbiome contributes to a healthy and strong epithelial barrier and a healthy immune system (120, 137). Not only the gut, but the lung has a microbiome as well. The lung microbiome reaches maturity two to three months after birth (138). Cross-colonization may take place between the lungs and the gut, with sputum containing lung bacteria being swallowed and small amounts of gastric fluids being aspirated into the lungs (5). The predominant phyla found in the lung microbiome are *Bacteroidetes* and *Firmicutes*, and the most common bacterial communities consist of *Streptococcus, Prevotella, and Veillonella* (139). HMOs can potentially support the health of the lung microbiome indirectly through cross-colonization by the gut microbiome, where the growth of commensal bacteria can be directly influenced by higher concentrations of HMOs (5).

Alternations in the respiratory microbiome during respiratory infections have been observed, specifically during RSV infection. The severity of an RSV infection might be related to changes in the respiratory microbiota (140). One study in 96 children for example showed an increase in diversity of the upper respiratory microbiome in children with severe RSV-induced bronchiolitis (141). HMOs can potentially reduce the severity of RSV infection by supporting the health of the lung microbiome.

The maturation and formation of a proper intestinal epithelial barrier and a proper lung epithelial barrier is important to protect against invading pathogens and to prevent sensitization to allergens (16). HMOs can exert direct effects on the gut epithelial barrier, as shown in *in vitro* studies. For example, LNnT, 2’FL, and 6’-sialyllactose (6’SL) are reported to dose-dependently induce maturation of the gut epithelium, as evidenced by growth inhibition and enhanced differentiation in HT-29, Caco-2, and HIEC cells (142–144). Other reports show that 3’siallylactose (3’SL), 6’SL, and 2’FL strengthen the gut epithelial barrier by promoting the production of mucus and the formation and strengthening of the tight junctions (144, 145).

Most of the HMOs reach the large intestine undigested. They are then metabolized by specific gut bacteria. The main resulting metabolites of this process are the SCFA, of which butyrate, acetate, and propionate are the best-known and most-studied (16, 146, 147). *B.infantis*, abundantly present in the infant gut microbiome, is especially efficient at metabolizing HMOs into SCFA (123). In an *in vivo* study in rats, it was shown that acetate can increase the expression of genes related to mucus production, as well as support the differentiation of goblet cells (148). In rats, it was shown that the expression of mucus-related genes was upregulated by butyrate as well (149). Another mechanism by which SCFA support the barrier integrity, is by increasing the expression of tight junction protein-related genes Zonula Occludens-1 (ZO-1) and Occludin, therefore maintaining and strengthening the TJs between ECs (149, 150).

Studies show that RSV and RV can disrupt the epithelial barrier in the lung (151, 152). Both HMOs and SCFA become systemically available and could thus directly affect the lung epithelial barrier (14, 16, 126). For example, the expression of tight junction proteins in an *in vitro* model of lung ECs was increased by butyrate and propionate (153). HMOs and SCFA might have similar effects on the lung epithelial barrier as on the gut epithelial barrier and thus support a strong lung epithelial barrier.

### 5.3 Direct or indirect modulation of innate and adaptive immune cells involved in viral infections

HMOs can interact with cells of the immune system directly and indirectly. Direct interaction takes place through various glycan-binding proteins (lectins) which are expressed on immune cells and ECs, allowing HMOs to directly influence both the epithelial barrier and the immune response. Various types of lectins exist and are relevant in the response to infections: galectins, sialic acid binding immunoglobulin-like lectins (siglecs), selectins, and C-type lectins (16, 147). Indirect interaction is mediated by HMOs metabolites, the SCFA (**Table 1**). The interactions between HMOs or SCFA with these receptors can also play a role in asthma prevention and treatment. These interactions and effects are described in section 6.2 and 6.3.

#### 5.3.1 Galectins

Galectins are known to interact with viruses and can both promote and inhibit viral infections (154, 155). Ligand-bound galectins either directly relay signals into the cell, or become soluble (156). Soluble galectins can act as ligands themselves and bind receptors on mucosal immune cells (157). Some HMOs bind to a selection of galectins, while 25 HMOs were found to bind galectin-1, -3, -7, and -9, albeit with varying affinities (158). Another group reported that from the studied galectin-1, galectin-2, galectin-3, galectin-4, galectin-7, and galectin-8, only galectin-2 did not bind HMOs. The galectins all recognized a unique binding site in the HMOs (159). Galectins are involved in the modulation of both the innate and adaptive immune response, and they can exert both inflammatory and anti-inflammatory effects (154, 155). The exact role of galectins during viral infections is not yet fully understood, and further elucidation is warranted.

#### 5.3.2 Siglecs

Siglecs are expressed on various antigen presenting cells (APCs), basophils, eosinophils, NK cells, and mast cells, these are cell types that are involved in either viral defense and/or allergic responses (160, 161). The Siglec family consists of 15 members, of which some contain immunoreceptor tyrosine-based inhibitory motif (ITIM), a regulator of immune responses (146, 161). So far, only 3’SL and 6’SL have been found to bind to siglec-1, siglec-5, siglec-7, siglec-9, and siglec-10 (16). Siglec-1 was the first identified siglec and therefore the most studied. Siglec-1 expression on myeloid cells is induced by IFNγ and is thought to play an important role in the initiation of an immune response to viral infections (162). Viruses can however also interact with siglec-1 to downregulate the immune response. RV can for example inhibit the ability of DCs to stimulate T cells by inducing the expression of PD-L1 and siglec-1 (163). Inhibition of siglec-1 with a monoclonal antibody reversed the inhibitory phenotype. 3’SL and 6’SL can bind siglec-1, but the effect of binding is unknown. 3’SL and 6’SL could have a similar effect as a monoclonal antibody if the binding blocks the binding site of siglec-1 (16, 163).

#### 5.3.3 Selectins

Pro-inflammatory cytokines induce the expression of selectins on ECs. Selectins form a family of cell adhesion molecules which are essential for the first stages of leukocyte trafficking in inflammation (146). Sialylated HMOs (3’SL and 3’-sialyl-3-fucosyl-lactose (3’S3FL)) have been shown to interact with selectins, which inhibits the adhesion of monocytes, lymphocytes, and neutrophils to human umbilical vein endothelial cells (164, 165). The reduced influx of immune cells can possibly prevent severe inflammation in the lungs (147).

#### 5.3.4 C-type lectins

The last group of lectins interacting with HMOs are the C-type lectins. Four subgroups of C-type lectins exist: the sialo-glycoprotein receptor family, the dectin-1 subfamily of asialo glycoprotein receptors, the DC immune receptor subfamily, and the Mannose receptor family (146, 166). C-type lectins are expressed by APCs, where they act as PRRs, and thus play an important role in the regulation of immune responses to pathogens. They are involved in the internalization of antigens and the subsequent antigen presentation (146, 167). Some C-type lectins contribute to the transmission of viruses. Dendritic cell-specific intercellular adhesion molecule 3 (ICAM-3) grabbing non-integrin (DC-SIGN) for example aids in the transmission of several different types of viruses. One study suggested that RSV binding to DC-SIGN inhibits part of DC activation and might thus limit the antiviral response via this mechanism (168). Moreover, evidence exists that DC-SIGN signaling induced by fucose-expressing pathogens or viruses induces a type 2 immune response (169). A study showed that 2’FL, as well as a mix of HMOs from pooled human milk, bind DC-SIGN and prevent other ligands from binding (170). Preventing the binding of other ligands might prevent the inhibition of DC activation by RSV and could thus possibly contribute to the antiviral immune response. It is however unknown whether binding of DC-SIGN by HMOs has a biological effect, or simply blocks the receptor.

#### 5.3.5 Toll-like receptors

TLRs are important for the recognition of viruses (54). Evidence suggests that, besides lectins, HMOs are also able to bind TLRs. Some HMOs activate while other HMOs inhibit TLR signaling. Lacto-N-triose II (LNT II) was for example found to activate all TLRs, while 3FL was found to only activate TLR2. 2’FL, 3FL, 6’SL, and LNnT on the other hand were found to inhibit TLR5 and TLR7 signaling. 3FL additionally inhibited TLR8. Both the activation and inhibition of TLRs by HMOs are thought to contribute to immune balance (171–173).

### 5.4 Preclinical evidence

The effects of HMOs on viral infections have been tested in animal models as well. In a murine model of influenza infection for example, mice treated with 3FL prior to infection had an enhanced anti-viral response and an increased survival rate (174) (**Table 2**).

Next to the direct interactions of HMOs with immune cells, indirect effects of HMOs are possible via metabolites, such as SCFA, resulting from the fermentation of HMOs. SCFA are known to have effects on various immune cells. SCFA can increase the activation of anti-inflammatory immune cells, such as Treg cells, and can increase the production of regulatory cytokines (175, 176). On top of this, SCFA have been shown to directly interact with DCs and T cells via the G protein-coupled receptors (GPRs) present on the cell surface, further increasing their ability to modulate immune responses (177) (**Table 2**).

The effect of SCFA has been assessed in animal models as well. In an *in vivo* murine model of RSV infection, Antunes et al. reported that oral administration of acetate protected the mice from RSV infection (178). The anti-viral effects observed involved the activation of GPR43 and increased expression of interferon-stimulated genes in the lungs, which subsequently led to lower viral load. The effect of acetate was ameliorated in *GPR43-/-* mice, supporting the involvement of GPR43 in antiviral pathways in the lungs (**Table 2**).

### 5.5 Clinical evidence

Several studies on the protective effects of specific HMOs on viral infections have been performed, however, clinical evidence on respiratory viral infections is still very limited. One study, for example, followed mother-infant pairs for 24 weeks and showed that higher levels of lacto-N-fucopentaose II (LNFPII) in the human milk were associated with lower incidence of respiratory problems, such as infection or wheezing, after 6 weeks of life (179). In an intervention study, the effects of 2’FL and LNnT were studied in infants receiving formula for 6 months. Supplementation of the infant formula with 2’FL and LNnT was associated with lower parent-reported episodes of bronchitis, suggesting that these particular HMOs have a possible protective effect on the lungs (180). In another intervention study with prebiotics (GOS polydextrose mixture), probiotics, or placebo, infants in the prebiotics and probiotics groups had a lower incidence of viral respiratory tract infections compared to the infants receiving a placebo, with RV being the most common infection. The severity of the RV infections between all groups was similar (181).

More general effects of HMOs on inflammatory markers have been observed in infants as well. For example, healthy infants receiving formula milk supplemented with GOS in combination with 2’FL had 29% to 83% lower concentrations of pro-inflammatory cytokines (IL1ra, IL6, IL1β, TNFα, and IFNγ) in their blood compared to infants receiving formula milk GOS alone. The cytokine levels of infants fed with 2’FL supplemented formula were similar to infants fed human milk. In the same study, peripheral blood mononuclear cells (PBMCs) were stimulated *ex vivo* with RSV, and similar results were found. PBMCs from formula-fed children secreted higher levels of pro-inflammatory cytokines than PBMCs from breastfed infants and PBMCs from infants fed with the 2’FL supplemented formula (182) (**Table 2**).

## 6 Possible connection between HMOs and asthma prevention

### 6.1 Modulation of the microbiome and epithelial barrier

An altered gut microbiome has been associated with the development of asthma (183). The gut microbiota can be modified using HMOs (124). Several bacterial strains have shown beneficial effects in promoting intestinal epithelial barrier properties and/or lowering inflammatory responses in intestinal epithelial cells (144, 145).

Studies have shown a disrupted lung epithelial barrier in asthma, which is associated with a decreased expression of the TJ proteins occludin and ZO-1 (184, 185). Some allergens have proteolytic activity, which can damage the lung epithelial barrier and allow easier interaction of allergens and pathogens with mucosal DCs. In addition, the type 2 inflammatory response in the bronchial mucosa can affect the epithelial barrier of the lungs, as IL4 and IL13 are known to downregulate TJ expression (186). Moreover, IL4 and IL13 also activate Notch-signaling, promoting differentiation of airway basal cells into mucus producing goblet cells, and thus leading to goblet cell hyperplasia and mucus plugging (187, 188).

HMOs have been shown to promote barrier integrity in the gut and could have similar effects on the lung epithelial barrier through their systemic availability or via metabolites resulting from their fermentation in the gut (e.g. SCFA) (144, 145). Moreover, in an *in vitro* study using BECs from asthmatic patients, it was shown that the epithelial barrier was restored with the addition of (histone deacetylase) HDAC inhibitors (184). The SCFAs become available in low concentrations systemically and could have similar effects in the lungs, as they are HDAC inhibitors as well (189).

### 6.2 Direct or indirect modulation of innate and adaptive immune cells involved in asthma development and/or symptoms

Since HMOs are glycan-type structures, they can bind to various glycan-binding receptors present on structural and immune cells. HMOs may directly affect immune cells by interacting with these glycan receptors. Additionally, they may indirectly influence the immune system by modulating the levels of soluble glycan receptors or SCFAs. Below, the potential implications of HMOs in preventing allergic sensitization are outlined first, followed by their direct or indirect effects on the chronic inflammatory response in established asthma, based on *in vitro* and *in vivo* studies. The receptors described may also play a role in the protection against viral infections, which is described in section 5.3 (**Tables 1**, **2**).

#### 6.2.1 Allergic sensitization cascade and cells bridging the innate and adaptive immune response

As previously mentioned, in allergic asthma, allergen-induced type 2 activation of BECs leads to activation of ILCs and DCs. DCs drive allergen specific Th2 immunity in the afferent lymph nodes, where B cells are instructed for IgE isotype switching. HMOs are thought to support a proper balance between Treg and Th1 versus Th2 immune responses by modifying DC function. For example, treatment of human monocyte derived DCs (moDCs) with HMOs mixtures from pooled human milk increased the secretion of the tolerogenic cytokines IL10 and IL27, while lowering the secretion of inflammatory cytokines TNFα and IL6. Furthermore, when these moDCs were co-cultured with naive T cells, the moDCs induced the differentiation of naïve T cells into Treg cells (172). In transwell co-cultures using IECs and activated PBMCs, a mixture of 2’FL and short-chain (sc) GOS and lcFOS enhanced the secretion of IFNγ and/or galectin-9 after exposure to CpG, while decreasing IL13 (190). In models where IECs were co-cultured with moDCs and moDCs with naïve CD4+ T cells, 3FL enhanced the secretion of IL10 and IL17, while decreasing the production of IL12p70, IL13, and IL23 in response to ovalbumin (OVA). Furthermore, 3FL decreased the differentiation of naïve T cells into Th2 cells, while inducing differentiation into Treg cells. Moreover, 3FL silenced the Th2 effector response (191). Even though these effects were observed in mucosal *in vitro* models using IEC, HMOs may also be capable of immunomodulation at the mucosal surface of the lungs.

#### 6.2.2 C-type lectins

HMOs become systemically available, and may therefore act on BEC as well. C-type lectin receptors have been shown to be involved in the modulation of the DC and T cell responses by HMOs. HMOs can interact with C-type lectin DC-SIGN (16, 167). Allergens binding DC-SIGN activate DCs, and DC-SIGN is thus thought to be involved in the sensitization phase of allergic diseases (192). For example, DC-SIGN was found to be involved in the recognition and uptake of the house dust mite (HDM) allergen der p 1 (193). As indicated previously, evidence exists that DC-SIGN signaling induced by fucose-expressing pathogens or viruses induces a type 2 immune response (169). This might be prevented by HMOs binding to DC-SIGN. Moreover, the binding of DC-SIGN by HMOs could potentially prevent the binding and uptake of allergens by DC-SIGN, thereby preventing DC maturation and possibly activating tolerogenic responses via DC-SIGN. Furthermore, one study has shown that a mix of HMOs isolated from pooled human milk was able to reduce the expression of DC-SIGN on DCs, which would limit DC activation and subsequent allergic sensitization (172).

### 6.3 Effector cells in chronic allergic airway inflammation and tissue remodeling

In allergic asthma, the binding of airway allergens to IgE opsonizing bronchial mast cells leads to IgE receptor crosslinking, resulting in airway narrowing via smooth muscle contraction, mucus hypersecretion, and bronchial wall edema. The activity of mast cells, therefore, plays an important role in the development of symptoms in allergic asthma. Beneficial bacteria like *Lactobacillus* spp. were able to suppress the genes encoding the FcϵR and histamine receptors *in vitro* (194). The HMO fucosyl-α1,3-GlcNAc (3FN) has been shown to increase the abundance of *Lactobacillus* spp. in bacterial cultures derived from infant stool samples (195). *Lactobacillus* spp. produce several fermentation products, including the SCFAs propionate and butyrate. Propionate and butyrate have been shown to inhibit mast cell activation through modulation of FcϵRI-mediated signaling (153). The results from these studies suggest that *Lactobacillus* spp. might contribute to the inhibition of mast cell activation possibly through the production of SCFA, which in turn suppress genes responsible for FcϵRI-mediated signaling. Similarly, the abundance of *E. faecalis* is found to positively correlate with the intake of HMOs, and these bacteria inhibit degranulation of bone-marrow derived mast cells *in vitro* (196).

In chronic asthma, a Th2-driven chronic inflammation and eosinophilic airway inflammation contribute to airway hyperresponsiveness, goblet cell metaplasia, tissue damage, and tissue remodeling. Inflammatory macrophages and Tc2 cells contribute via additional secretion of type 2 cytokines IL4 and IL13 (41, 45). The effects of HMOs on these types of cells are currently largely unknown. However, HMOs may be able to modify the responses of these effector cells in chronic inflammation via several types of (soluble) glycan receptors.

#### 6.3.1 Galectins

Various HMOs were shown to bind galectin-1 (158). Galectin-1 has a regulatory role in allergic asthma. T cell homeostasis is maintained by galectin-1, by inducing the production of IL10 by T cells, while suppressing the release of TNFα and IFNγ, and supporting the functions of Treg cells (197–199). These findings are further supported by a murine study, in which galectin-1 deficient mice developed more severe inflammation in response to allergen challenge when compared with wild-type mice (200). Galectin-1 is furthermore able to inhibit eosinophil migration, reducing the number of eosinophils in the lungs and lowering inflammation (201). Moreover, a study in a murine model of AAI showed that administration of galectin-1 inhibited the recruitment of immune cells to the lungs, as well as the secretion of cytokines and mucus (202). HMOs could modulate the release of galectin-1 and support immunomodulatory properties of galectin-1 (**Table 1**).

**Table 1:** The effects of interactions between HMOs and receptors, and SCFA and receptors.

HMOs				
HMO	Model	Receptor	Effect	Reference
2’FL	Binding assay	Galectin-1, -3, -4, -7 and -8		(158, 159)
	OUW-SIGN cells	DC-SIGN	Prevent binding of other ligands	(170)
	THP-1 reporter cell line and HEK reporter cell line	TLR5, TLR7	Inhibition of TLR5 and TLR7 activation.	(171)
	RAW264.7 cells	TLR4	Dose-dependent inhibition of TLR4/NF-κB pathway, upregulation miR-146a expression	(221)
3FL	THP-1 reporter cell line and HEK reporter cell line	TLR2, TLR5, TLR7, TLR8	Activation TLR2, inhibition TLR5, TLR7, TLR8; contribute to immune balance	(171)
3’SL	Binding assay	Siglec-1, -3, -5, -7, -9, -10		(159, 246)
	Autologous human moDC-T cell co-culture	Siglec-1	May enhance immune response to RV infection	(163)
	Monocytes, lymphocytes, neutrophils migration over HUVEC	Selectins	Inhibition adhesion monocytes, lymphocytes, neutrophils to human umbilical vein endothelial cells	(164, 165)
	Murine model of AAI	Galectin-1, -8	Involved in modulation of adaptive and innate immune response, both pro-inflammatory and anti-inflammatory. Effect of HMO binding not yet studied	(158, 159)
6’SL	Binding assay	Siglec-1, -3, -5, -7, -9, -10		(159, 246)
	Autologous human moDC-T cell co-culture	Siglec-1	Possibly enhance immune response to RV infection	(163)
	THP-1 reporter cell line and HEK reporter cell line	TLR5, TLR7, TLR8	Inhibition of TLR5 and TLR7 activation. Synergistic effect on ssRNA40 induced TLR8 activation.	(171)
3’S3FL	Monocytes, lymphocytes, neutrophils migration over HUVEC	Selectins	Inhibition adhesion monocytes, lymphocytes, neutrophils to human umbilical vein endothelial cells	(164, 165)
LNT II	THP-1 reporter cell line and HEK reporter cell line	TLR2, TLR3, TLR4, TLR5, TLR7, TLR8, TLR9	Dose-dependent activation of all TLRs. NF-κB dependent IL10 and TNFα secretion.	(171)
LNnT	Binding assay	Galectin-1, -3, -4, -7 and -9		(158, 159)
	THP-1 reporter cell line and HEK reporter cell line	TLR5, TLR7	Inhibition of TLR5 and TLR7 activation.	(171)
LNFP I	Binding assay	Galectin-1, -3, -4, -7 and -9		(158, 159)
HMOS	Human moDC-naïve CD4+ T cell co-culture	TLR4, DC-SIGN	Increased regulatory and decreased type 1 moDC and T cell responses during LPS exposure, mediated via HMOS binding to TLR4 and DC-SIGN	(172)
	OUW-SIGN cells	DC-SIGN	Preventing binding of other ligands	(170, 172)

SCFAs				
SCFA	Model	Receptor	Effect	Reference
Acetate	Murine model of RSV infection	GPR43	Activation of GPR43, increase IFN-β production, protect against RSV infection in mice	(178)
	Murine model of AAI	GPR43	Activation of GPR43, reducing recruitment of immune cells	(225)
	Murine model of AAI	HDAC9	Increase in Foxp3 acetylation, upregulation of number of Treg and increasing their function	(177)
Butyrate	Murine model of AAI	HDAC	Inhibit cytokine production of ILC2, as well as proliferation ILC2	(229)
	Specific pathogen-free, microbiota -deficient, and germ-free mice	histone H3 acetylation	Upregulation of FoxP3 expression and subsequent increased number of Treg cells	(228)
Propionate	Murine model of AAI	GPR41	Protection against HDM-induced AAI. Decreased recruitment of eosinophils to the lungs. Altered DC function, resulting in decreased Th2 effector function	(132)

Galectin-3, on the other hand, appears to have a pro-inflammatory role in allergic asthma, as a study in mice showed that galectin-3 expressing inflammatory cells were recruited to the lungs upon allergen exposure (203). Furthermore, the levels of galectin-3 were found to be upregulated on the cell surface of eosinophils derived from allergic subjects, and they had increased adhesive interactions (201). A murine study showed that mice deficient in galectin-3 had a decrease in eosinophilic infiltration of the lungs, as well as a dampened development of a Th2 inflammatory response in the lungs and a reduction in airway remodeling (204). Depending on the cell type, galectin-3 expressed on the cell surface is mostly involved in cell activation, adhesion, migration, and apoptosis. HMOs can bind various galectins, including galectin-3. Blocking galectin-3 could influence these processes and possibly reduce immune cell migration toward the lungs (**Table 1**) (16, 205, 206).

Galectin-9 is evidenced to have both a pro-inflammatory and an anti-inflammatory role in allergic asthma. In models of allergic asthma in mice and guinea pigs, higher expression of galectin-9 in the lungs was related to an increase in the recruitment of eosinophils (207, 208). However, in a model of AAI in guinea pigs, exogenously administered galectin-9 was shown to reduce recruitment of eosinophils to the lungs and suppress airway resistance (209). Another study showed that galectin-9 could be involved in the regulation of activated eosinophils, as galectin-9 induced apoptosis in activated eosinophils, but not in non-activated ones (210). Furthermore, *in vitro* studies have shown that galectin-9 can bind IgE, which prevents the formation of IgE-allergen complexes and thus the degranulation of mast cells (209). Moreover, low levels of galectin-9 were found to be associated with lower levels of Tregs and tolerogenic DCs in the blood and intestinal biopsies of food-allergic individuals. After DCs were exposed to galectin-9, their ability to generate Treg cells was restored (211). Similar results were found in a mouse model of food allergy, where galectin-9 levels were found to be increased after dietary intervention with GOS/lcFOS and *B.breve*, which corresponded with decreased mast cell degranulation *in vivo*, as well as increased differentiation into Th1 and Treg cells *in vitro and in vivo* (212). More studies into the role of galectin-9 are however needed to determine whether similar mechanisms play a role in allergic asthma and whether HMOs could potentially influence galectin-9 levels in a similar fashion as GOS/lcFOS.

#### 6.3.2 Siglecs

Siglec-7 and siglec-8 were shown to be involved in allergic immune responses, possibly playing a role in allergy and allergic asthma (213–215). Sialic acid containing HMOs, such as 3’SL and 6SL, can bind siglec-7 and siglec-8 with low affinity (16). Siglec-7 is expressed on various immune cells, including monocytes, eosinophils, and mast cells. Activation of siglec-7 with a humanized antibody prevented the degranulation of human cord blood-derived IgE-sensitized mast cells (216). The gene expression of siglec-8 is increased in the sputum cells of asthmatic individuals. Siglec-8 is expressed on the surface of both eosinophils and mast cells and, similar to siglec-7, crosslinking siglec-8 with a humanized antibody prevented the degranulation of mast cells *ex vivo* (214). HMOs could potentially exert a similar effect on siglec-7 and siglec-8 as an antibody, preventing the degranulation of mast cells.

### 6.4 Pre-clinical evidence

In various murine studies, Th2 skewing was reduced, and intestinal Treg numbers increased by treatment with *Bifidobacterium*, and *Lactobacillus* spp supplementation (217, 218). HMOs support the colonization of *Bifidobacterium* and might thus exert its protective effects via modulation of Th skewing (122). A study performed in a murine model of HDM allergic asthma revealed that both 2’FL and 6’SL at biologically relevant doses decreased the amount of circulating IgE, decreased levels of IL4 and IL6 in the lungs, and decreased inflammatory cell infiltration in the lungs. Moreover, increased levels of *Bacteroidetes* and *Clostridia* were found, as well as increased levels of SCFA in both the intestine and the blood (219). Studies on the effects of HMOs in food allergy can be relevant for understanding the effect of HMOs in allergic asthma, as the involved immunological mechanisms are thought to be comparable. One study for example showed that mice, previously intragastrically sensitized to OVA, had increased airway inflammation after intranasal exposure to both OVA and HDM. These results show a connection between intestinal sensitization, and thus a break in oral tolerance, and allergic asthma (220). In a murine cow’s milk allergy model, 2’FL specifically reduced the secretion of allergen-specific IgE, mast cell degranulation, and the levels of TNFα, IL4, and IL6 in serum. These results are similar to what was observed in an HDM allergic asthma model using 2’FL. Further *in vitro* studies revealed that 2’FL directly inhibits TLR4, which plays an important role in allergy (221). Similar findings were obtained in OVA-sensitized mice after daily treatment with 6’SL. An increase in Treg cells was found in the Peyer’s patches and mesenteric lymph nodes in the 6’SL treated mice as well (222). Yet another study showed that mice treated with 3FL during OVA sensitization had decreased Th2 activation, while Treg numbers increased. The administration of 2’FL had beneficial effects as well, resulting in decreased mast cell activation (223). These studies provide evidence for the anti-inflammatory role that HMOs can play in allergy.

SCFAs can interact with various receptors, such as GPR43, GPR41, and GPR109A (177, 178). GPR43 and GPR41 are expressed on a wide variety of cells, including IECs, BECs, and immune cells. GPR109A is expressed on immune cells as well. All three major SCFA can bind GPR41, while only acetate and propionate can bind GPR43, and only butyrate can bind GPR109A (224). In a murine asthma model, it was shown that GPR43 is involved in protective mechanisms in asthma. OVA-sensitized *Gpr43-/-* mice had unresolved or aggravated inflammation, which appeared to be due to an increase in the recruitment of immune cells to the lungs (225). In another study, mice fed a high-fiber diet had increased concentrations of SCFA in their blood. This increased tolerogenic DC precursor numbers in the bone marrow, which in turn resulted in reduced HDM induced allergic inflammation in the lungs. In the same study, it was shown that the administration of propionate via drinking water protected against HDM-induced AAI as well, in a GPR41-dependent manner. This effect was mostly due to a decreased recruitment of eosinophils to the lungs (132). Activation of GPR43 and GPR41 by propionate could thus possibly have a protective effect on inflammation in asthma by promoting a tolerogenic DC phenotype and reducing the recruitment of immune cells, pointing to a potential role of HMOs as a source for SCFA production.

Furthermore, SCFA interact with histone deacetylases (HDACs) and therefore modify the expression of several genes. HDACs are regulators of T cell differentiation, and several HDACs, such as HDAC1 and HDAC9, have been found to be upregulated in allergic asthma (184, 226). Butyrate and propionate have been shown to inhibit HDACs in immune cells in mice, inducing the upregulation of Foxp3 and IL10 secretion, and the subsequent differentiation into Treg cells (227, 228).

The inhibitory effect of SCFA on HDACs has been studied in murine models of asthma. One study showed that the administration of butyrate via drinking water reduced airway inflammation in an ILC2 driven asthma model in mice, by inhibiting the proliferation and cytokine production of ILC2s, likely via HDAC inhibition (229). Another study showed that administration of acetate increased Foxp3 acetylation through HDAC inhibition, which upregulated the number of Treg cells, as well as increased their suppressive function. The induction of Treg cells was associated with the suppression of AAI symptoms (177). Yet another study showed that administration of 2’FL and 6’SL reduced ILC2 mediated airway inflammation in mice. This effect was shown to be due to increased SCFA levels (230).

### 6.5 Clinical evidence

Meta-analyses show that breastfeeding can reduce the risk of allergic asthma development in children (17, 18). The rich and varying composition of human milk poses challenges in linking breastfeeding to allergy and asthma risk, but HMOs could be one of the main factors involved. The composition of human milk can affect the outcomes of linking breastfeeding to allergy risk. One study determined different profiles of maternal HMOs in a cohort of 620 babies. From these profiles, 7 classes were identified, based on the secretor and Lewis groups, as well as neutral and acidic groups present. Children receiving acidic Lewis HMOs human milk (e.g. high 3FL, 3’SL) had a higher risk of allergic disease and asthma up to 18 years of age compared to children receiving neutral acidic HMOs. The group fed with acidic predominant HMOs human milk (e.g. high 6’SL, low 3FL, 3’SL) however, had a lower risk of being sensitized to food allergens up to 18 years of age (231). In another study focusing on FUT2-dependent oligosaccharides, it was found that C-section-born children had a lower incidence of allergic disease at 2 years of age when fed with human milk containing FUT2-dependent oligosaccharides (232). Furthermore, a strong association between the levels of SCFA in infant’s feces and the development of asthma between 3 and 5 years of age was found. Children with high levels of butyrate and propionate in the feces were significantly less likely to develop atopic sensitization and subsequent development of asthma (233).

Current clinical studies give some evidence for the protective effects of breastfeeding on allergic diseases. More studies are needed to elucidate the influence of the composition of human milk on the effects on allergic diseases. Moreover, studies focusing specifically on the role of HMOs in asthma are needed as well, to better understand the presumed protective effects of HMOs.

## 7 The Gut-Lung axis

Besides the direct effects that HMOs and SCFA can have on the microbiome of the lung, they also affect the microbiome in the gut. The beneficial effects of a diverse, well-developed, and properly functioning gut microbiome may translate to a healthy development of the lungs as well, via the gut-lung axis. The gut-lung axis allows the interexchange of microbes and metabolic products of commensal bacteria to impact the lungs and vice versa. Microbiota can interchange via the trachea and gut tissue, while gut bacterial fermentation products, such as the short chain fatty acids (SCFA) acetate, propionate and butyrate, can enter the bloodstream and be transferred, albeit in low concentrations, to the bone marrow, and to the lungs where they can have anti-inflammatory effects (128, 132, 139). Evidence for the existence of a gut-lung axis comes from murine studies, as well as epidemiological studies. For example, germ-free mice were found to be highly susceptible to allergic airway disease (234). Similarly, depletion of the gut microbiome through antibiotic use in early life increases the risk of developing airway disease (235, 236). Furthermore, epidemiological studies point to an association between the use of antibiotics in early infancy, microbiome dysbiosis, and an increased risk of developing asthma (237, 238). A recent study in a murine HDM-induced allergic asthma model showed that this increased susceptibility might be related to the decreased systemic levels of indole-3-propionic acid (IPA), a bacterial metabolite. Mice treated with antibiotics had an increased susceptibility to HDM-induced allergic airway inflammation (AAI) and had a decreased level of IPA. While oral supplementation with IPA protected the mice from AAI (239). Moreover, another study demonstrated that inflammation in the gut predisposed mice to the development of allergen-specific airway response (220).

Beyond the interchange of microbes and metabolic products, Tregs generated in the gut are known to migrate to the lungs and vice versa (240). These migrating Tregs have been shown to reduce Th2 allergic responses in the lungs of allergic individuals (240). Lastly, antigens can travel from the gut to the lung and the other way around via the bloodstream (241). The lung can also influence the gut, as shown in mice, where respiratory influenza infections resulted in an altered intestinal microbiome, as well as intestinal injury (242). Furthermore, chronic pulmonary disorders are associated with symptoms of the gastrointestinal tract as well (243, 244). In early life, the composition and function of the microbiota can be shaped via breastfeeding (124). HMOs can function as prebiotics for beneficial bacteria and can in addition to this also directly impact immune function.

## 8 Discussion

In this review we described asthma as a common immune disease with an increasing prevalence worldwide (3). It is unknown how asthma exactly develops, but it is known to be associated with several risk factors, such as a genetic predisposition and a severe viral infection in early life, among others (1, 6).

Viral infections can increase the risk of developing allergic asthma by damaging the lung epithelial barrier (58–60). The lung epithelial barrier can be damaged by apoptotic cell death and TJ degradation (59, 62, 63). Allergens can cross the damaged lung epithelial barrier more easily, which increases the risk of sensitization (60). In addition, apoptotic ECs produce alarmins, which play a key role in the allergic response (64, 65).

On top of damaging the lung epithelial barrier, viral infections can cause a type 2 immune reaction, which can increase the risk of developing asthma as well (58–60). A type 2 skewed immune system could be more easily sensitized to allergens, especially if the lung epithelial barrier is also damaged. The combination of a damaged epithelial barrier and a type 2 skewed immune system might explain how severe viral infections increase the risk of allergic asthma.

**Figure 3:**
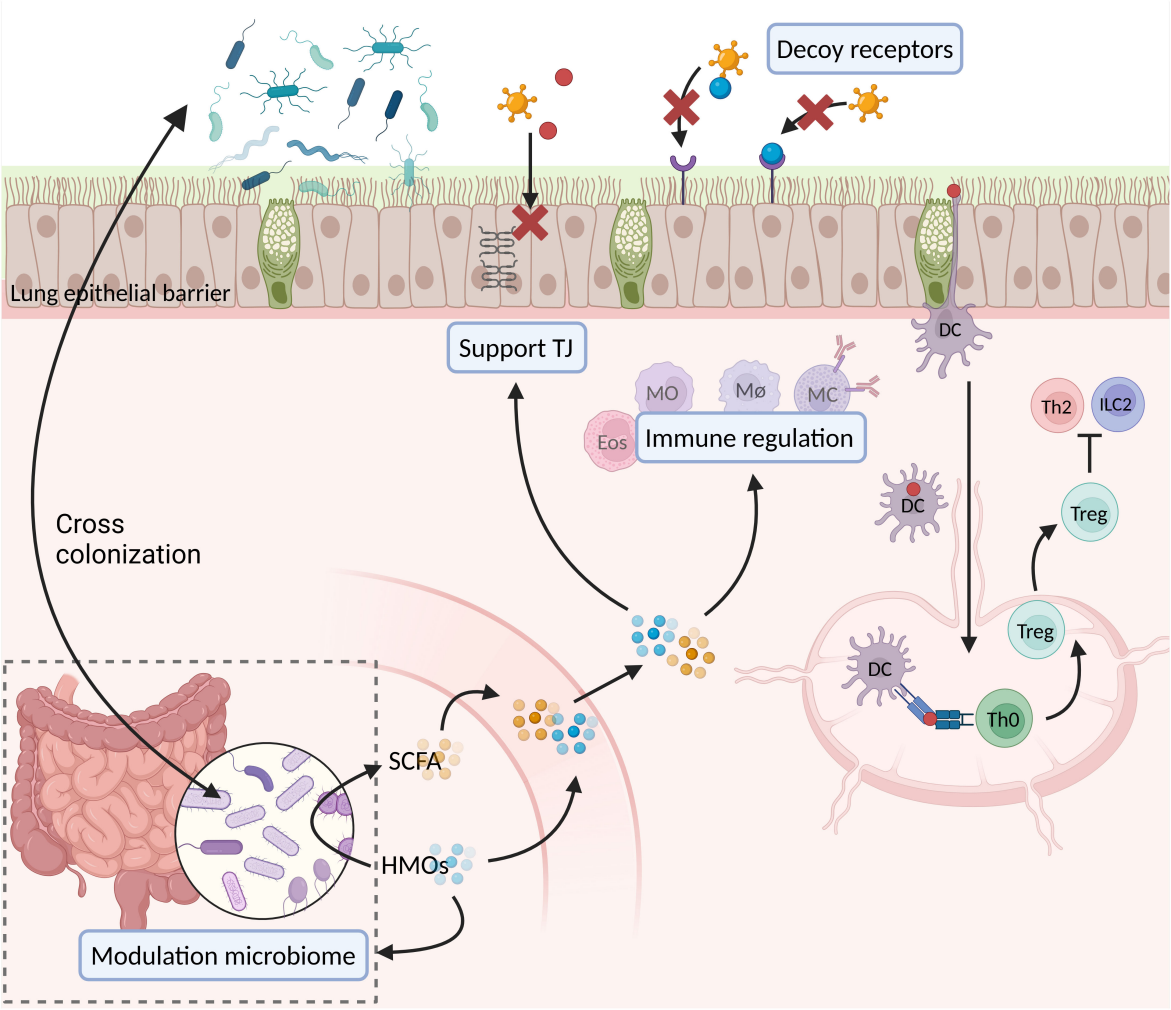
Suggested effects of HMOs and SCFA during viral infections and allergic sensitization. DC, dendritic cell; Eos, eosinophil; HMOs, human milk oligosaccharides; ILC2, group 2 innate lymphocyte cell; MC, mast cell; MØ, macrophage; MO, monocyte; SCFA, short chain fatty acids; Th0, naïve T helper cell; Th2, T helper 2 cell; TJ, tight junctions; Treg, T regulatory cell (created in BioRender.com).

Breastfed infants have a lower risk of developing a severe viral infection and/or asthma than formula fed infants, according to epidemiological data (245). HMOs might directly play an important role in this protective effect, but might also indirectly play a role via specific SCFAs (16, 146, 147). Directly, HMOs might strengthen the lung epithelial barrier (**Figure 3**). A strong lung epithelial barrier protects against invading pathogens and sensitization against allergens. HMOs can strengthen the gut epithelial barrier and possibly also exert similar strengthening effects on the lung epithelial barrier, as HMOs become systemically available in low concentrations (126, 142–144). HMOs might furthermore have protective effects through directly interacting with immune cells through glycan-binding proteins (**Figure 3**) (16, 147). Indirectly, SCFA might also strengthen the lung epithelial barrier (**Figure 3**). SCFA also strengthen the gut epithelial barrier and also become systemically available in low concentrations (14, 16). Furthermore, SCFA can also interact with immune cells through glycan-binding proteins, and exert protective effects in this way (**Figure 3**).

**Table 2:** Models exploring the effects of HMOs.

HMO	Model	Effect	Reference
2’FL	16HBE	Reduced viral load of RSV	(135)
	Co-culture of HT-29 intestinal epithelial cells and PBMCs	Promoted Th1 function, reduced IL13, increased galectin-9	(190)
	Co-culture of HT-29 intestinal epithelial cells with moDCs and subsequent naïve Th cells	Decreased differentiation of naïve T cells into Th2 cells, while increased differentiation into Treg cells	(191)
	HT-29, Caco-2Bbe intestinal epithelial cells and fetal small intestinal epithelial crypt cells	Induced maturation of epithelium cells	(142–144)
	HT-29, Caco-2Bbe and Caco-2 intestinal epithelial cells	Promoted mucus production. Promoted formation of TJs.	(144, 145)
	Murine model of HDM allergy	Decreased levels of circulating IgE, decreased levels of IL4 and IL6 in the lungs, decreased inflammatory cell infiltration in the lungs. Increased levels of *Bacteroidetes* and *Clostridia* and increased levels of SCFA in intestines and blood	(219)
	Murine model of cow’s milk allergy	Reduced allergic symptoms. Reduced secretion allergen-specific IgE, reduced mast cell degranulation, reduced serum levels of TNFα, IL4 and IL6	(221)
	Healthy infants	Lower concentrations of pro-inflammatory cytokines in plasma. Lower levels of pro-inflammatory cytokines in response to *ex vivo* stimulation with RSV	(182)
3FL	Co-culture of HT-29 intestinal epithelial cells with moDCs and subsequent naïve Th cells	Lowering IL12p70, IL23, IL13, enhancing IL17 and IL10 secretion. Silenced Th2 effector response	(191)
3FN	Bacterial cultures derived from infant stool samples	Increased abundance of *Lactobacillus* spp.	(195)
3’SL	16 HBE cells	Reduced viral load of RSV	(135)
	HT-29, Caco-2Bbe and Caco-2 intestinal epithelial cells	Promoted mucus production. Promoted formation of TJs.	(144, 145)
6’SL	16 HBE cells	Reduced viral load of influenza A	(135)
	HT-29, Caco-2Bbe, Caco-2 intestinal epithelial cells and fetal small intestinal epithelial crypt cells	Induced maturation of epithelial cells. Promoted mucus production and formation of TJs.	(142–145)
	HDM mouse model of allergy	Decreased amount circulating IgE, decreased levels IL4 and IL6 in the lungs, decreased inflammatory cell infiltration in the lungs. Increased levels of *Bacteroidetes* and *Clostridia* and increased levels of SCFA in intestines and blood	(219)
LNnT	16 HBE cells	Reduced viral load of influenza A	(135)
	HT-29, Caco-2Bbe intestinal epithelial cells and fetal small intestinal epithelial crypt cells	Induced maturation epithelium cells	(142–144)
2’FL + LNnT	Healthy infants	Fewer parent-reported bronchitis episodes	(180)
2’FL + 6’SL	Mouse model of AAI	Decrease in number, proliferation, and cytokine production of lung ILC2. Reduced airway inflammation	(230)
Total mixture	Human moDC-naïve CD4+ T cell co-culture	Increased secretion IL10, IL27, lowered secretion TNFα, IL6. Induction of differentiation into Treg	(172)

To conclude, evidence exists for the interplay between viral infections, asthma, and HMOs. However, the mechanisms behind these relations remain unknown. How viral infections exactly increase the risk of developing asthma, how HMOs protect against viral infections and asthma, and how HMOs interact with the relevant immune cells remains to be elucidated. Further studies will provide valuable insights into these relations and allow for the further improvement of formula milk when breastfeeding is not possible.
